# Supplementary material for: Gadd45B Deficiency Drives Radio-Resistance in BRAFV600E-Mutated Differentiated Thyroid Cancer by Disrupting Iodine Metabolic Genes
Source: Cancers (Basel). 2025 Sep 30;17(19):3201. doi: 10.3390/cancers17193201 (PMC12523365; doi:10.3390/cancers17193201)
Supplement: Supplementary file 1 [file cancers-17-03201-s001.zip › cancers-3775761-supplementary.pdf]

Supplementary Information

Gadd45B deficiency drives radio-resistance in $BRAF^{V600E}$- mutated differentiated thyroid cancer by disrupting iodine metabolic genes

Authors: Shan Jiang[1, †], Zhiwen Hong[1, †], Qianjiang Wu[2, †], Rouhan A[1], Zhaobo Wang[1], Xue Guan[3], Xinghua Wang[1], Ari A. Kassardjian[4], Yali Cui[1], Tengchuang Ma[1, *]

Affiliations: [1] Department of Nuclear Medicine, Harbin Medical University Cancer Hospital, Harbin 150081, China.

[2] Institute of Cancer Prevention and Treatment, Harbin Medical University Cancer Hospital, Harbin 150081, China.

[3] Animal Laboratory Center, The Second Affiliated Hospital of Harbin Medical University, Harbin 150081, China.

[4] Department of Radiation Oncology, City of Hope, 1500 E. Duarte Rd, Duarte, CA 91010, USA

[*] Corresponding author: Tengchuang Ma

Email: matengchuang1988@126.com

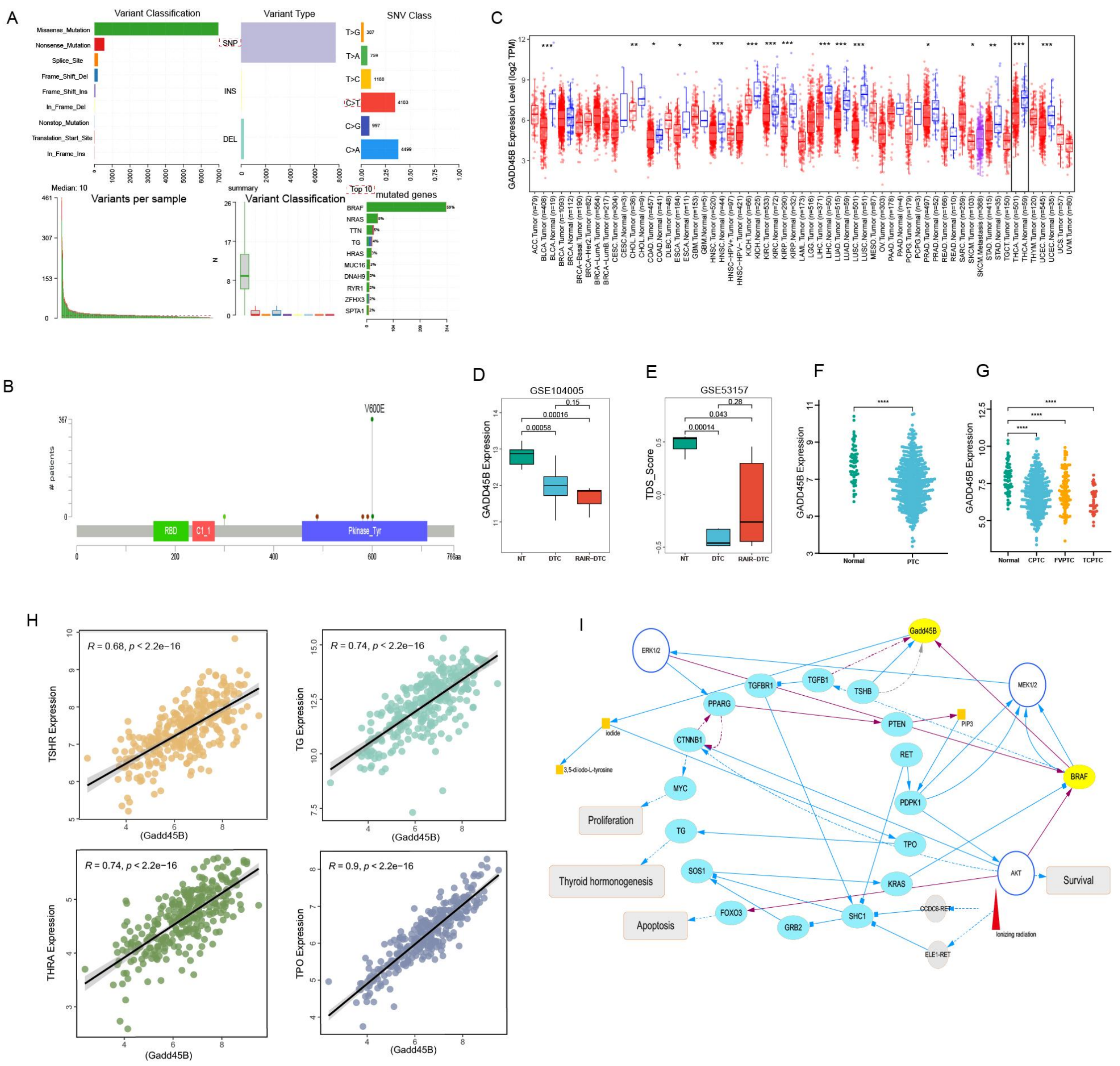

Figure S1

(A) Missense mutations are the most frequent mutation type, with a median mutation load in thyroid cancer.

(B) Distribution of BRAF mutations across protein domains in thyroid cancer (data sourced from cBioPortal).

(C) Gadd45B expression levels across pan-cancer datasets.

(D and E) Variability of Gadd45B expression levels and TDS scores during progression from normal tissue to DTC and RAIR-DTC (data collected from GSE104005 and GSE53157).

(F) Gadd45B expression levels in normal tissue versus papillary thyroid cancer (data sourced from TCGA).

(G) Gadd45B expression levels in normal tissue compared with different pathological subtypes of papillary thyroid cancer (data sourced from TCGA).

(H) Correlation between Gadd45B expression and iodine metabolism-related genes (*TSHR*, *Tg*, *THRA*, *TPO*) (data derived from TCGA).

(I) Network interaction between BRAF and Gadd45B in thyroid cancer (data sourcedfrom cBioPortal).Data are shown as mean ± SD. Statistical significance:ns: not significant, *$p < 0.05$, **$p < 0.01$, ***$p < 0.001$, **** $p < 0.0001$.

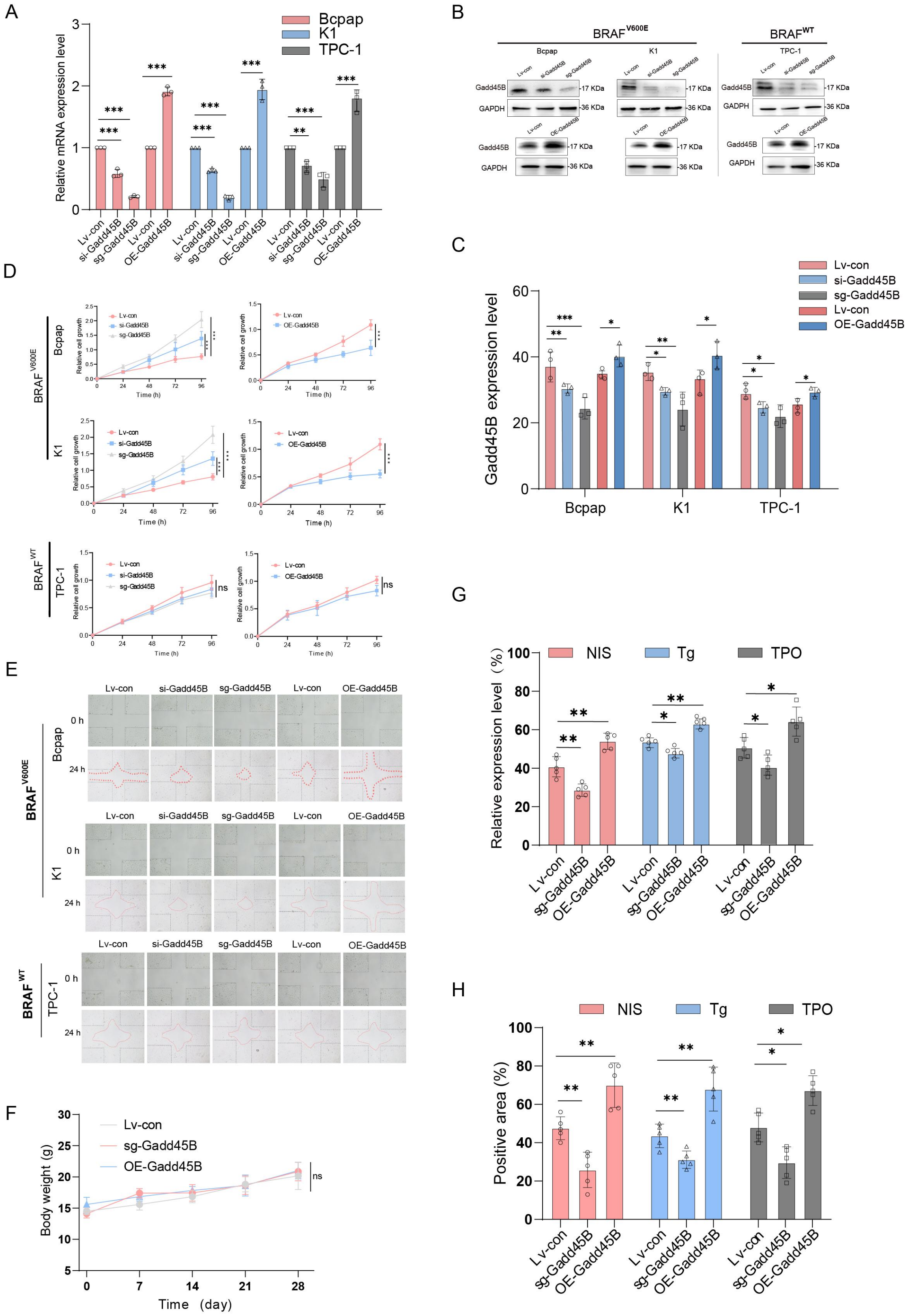

Figure S2

(A) mRNA expression levels and (B) protein expression levels of Gadd45B with (C) corresponding quantitative analysis in Bcpap, K1, and TPC-1 cell lines.

(D) Cell proliferation assays showing the effects of Gadd45B downregulation or overexpression in Bcpap, K1, and TPC-1 cell lines.

(E) Representative wound-healing assay images showing the effects of Gadd45B downregulation and overexpression in Bcpap, K1, and TPC-1 cell lines at 0 hours and 24 hours. Scale bar: 100 μm.

(F) Body weight curves of mice corresponding to Fig. 2L.

(G) Protein expression levels and (H) positive staining area of NIS, Tg, and TPO,corresponding to Fig. 2M and N.Data are shown as mean ± SD. Statistical significance: ns: not significant, *$p < 0.05$, **$p < 0.01$, ***$p < 0.001$, **** $p < 0.0001$.

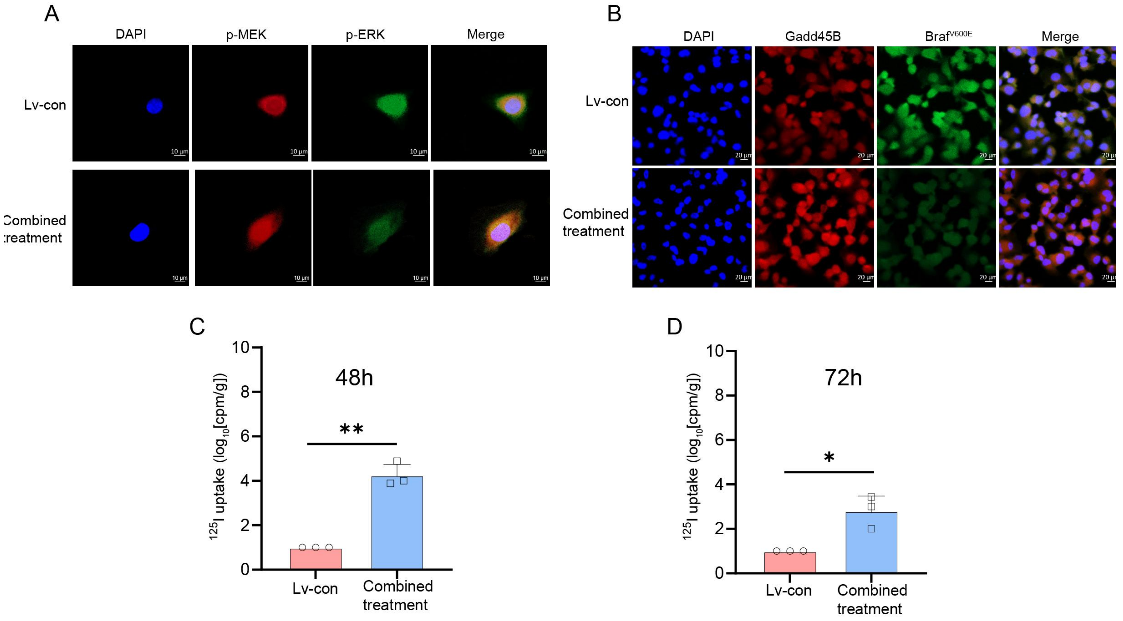

Figure S3.

(A) Representative immunofluorescence images showing the subcellular localization of p-MEK in K1 cells treated with combined Gadd45B overexpression, PLX4720, and AZD6244. Scale bar: 10 μm.

(B) Immunofluorescence images showing the subcellular localization of Gadd45B and BRAF^{V600E} in Bcpap cells following combined treatment. Scale bar: 10 μm.

(C) Quantitative analysis of RAI uptake levels at 48 hours *in vivo*.

(D) Quantitative analysis of RAI uptake levels at 72 hours *in vivo* (n=3 independentbiological replicates). Data are shown as mean ± SD. Statistical significance:ns: not significant *$p < 0.05$, **$p < 0.01$.

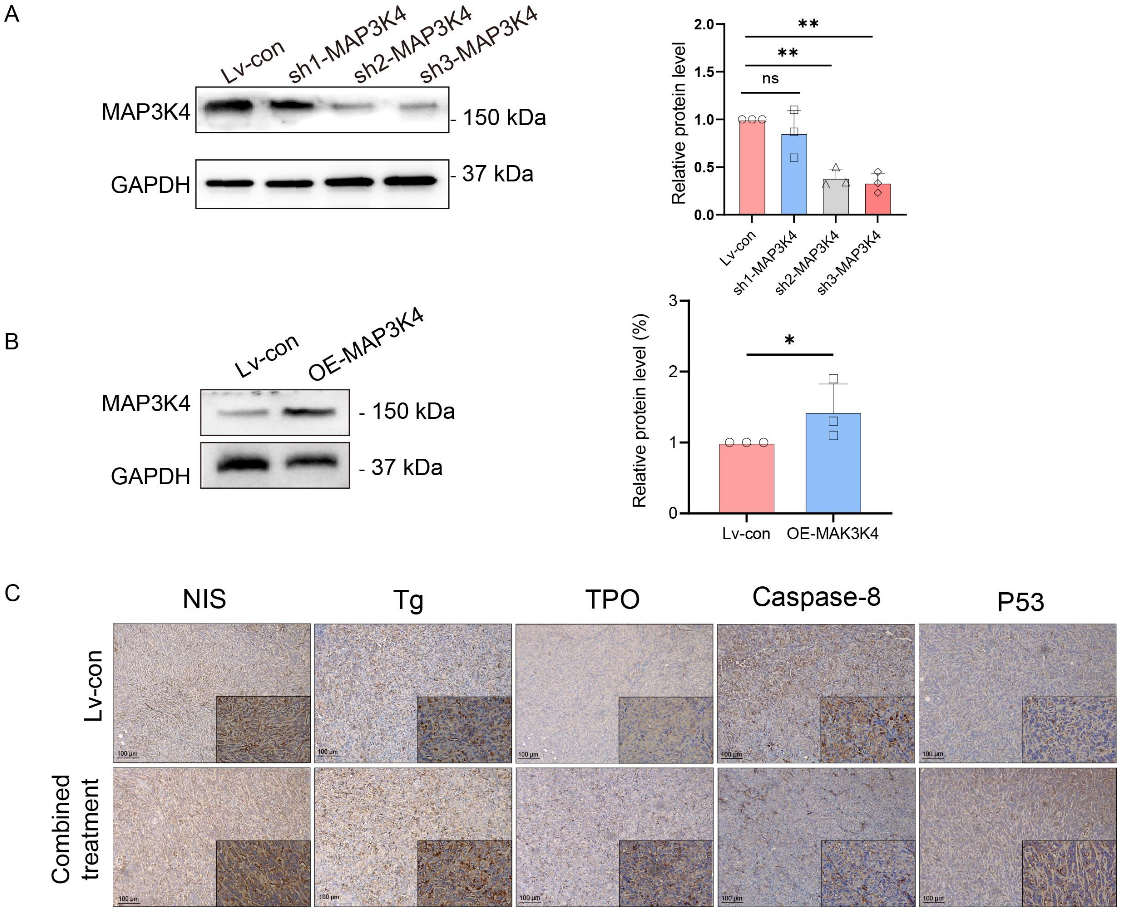

Figure S4.

(A) Western blot analysis and quantitative validation of *MAP3K4* knockdown efficiency (*n*=3 independent biological replicates).

(B) Western blot analysis and quantitative validation of *MAP3K4* overexpression efficiency (*n*=3 independent biological replicates).

(C) Representative IHC images showing the expression levels of NIS, Tg, TPO, caspase-8, and p53 in the combined treatment group. Data are shown as mean ± SD. Statistical significance:ns: not significant, *$p < 0.05$, **$p < 0.01$.

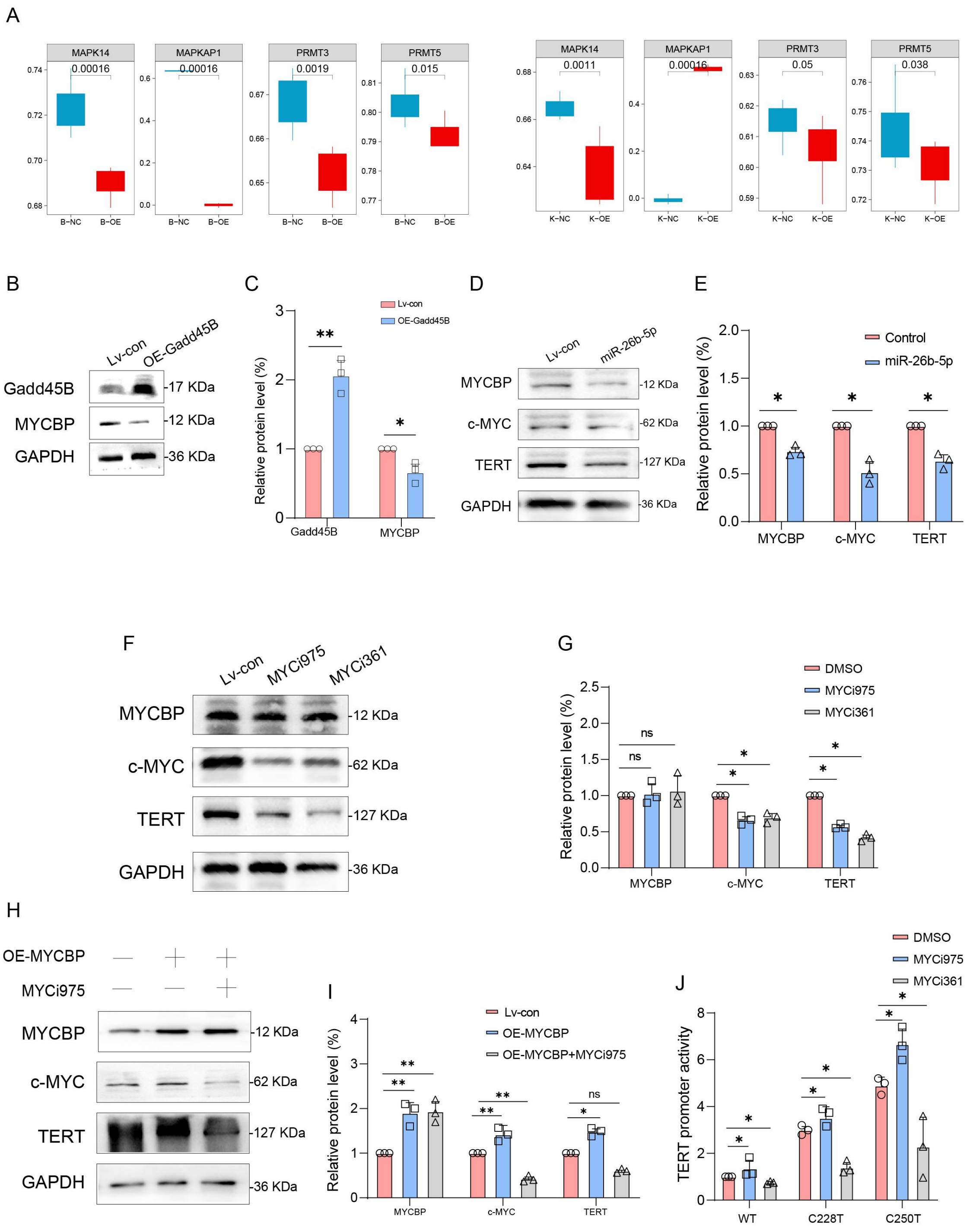

Figure S5.

(A) IP-MS analysis of Gadd45B in BRAF^{V600E}-mutated cell lines.

(B, C) *MYCBP* protein expression levels in Gadd45B-overexpressed Bcpap cells, with quantification (*n*=3 independent biological replicates).

(D, E) *MYCBP* and *c-MYC* protein expression levels in Bcpap cells transfected with 50

nM miR-26b-5p, with quantitative analysis (*n*=3 independent biological replicates).

(F, G) *MYCBP* and *c-MYC* protein expression levels in Bcpap cells treated with 3 μM MYCi975 or 1.5 μM MYCi361, with corresponding quantification (*n*=3 independent biological replicates).

(H, I) Effects of MYCi975 treatment on *MYCBP* and *c-MYC* expression in *MYCBP*-overexpressed Bcpap cells, with quantification (*n*=3 independent biological replicates).

(J) Dual-luciferase reporter assays for wild-type, C228T, and C250T-mutated *TERT* promoters treated with DMSO, MYCi975, or MYCi361 (*n*=3 independent biological replicates).Data are shown as mean ± SD. Statistical significance:ns: not significant *$p < 0.05$, **$p < 0.01$.

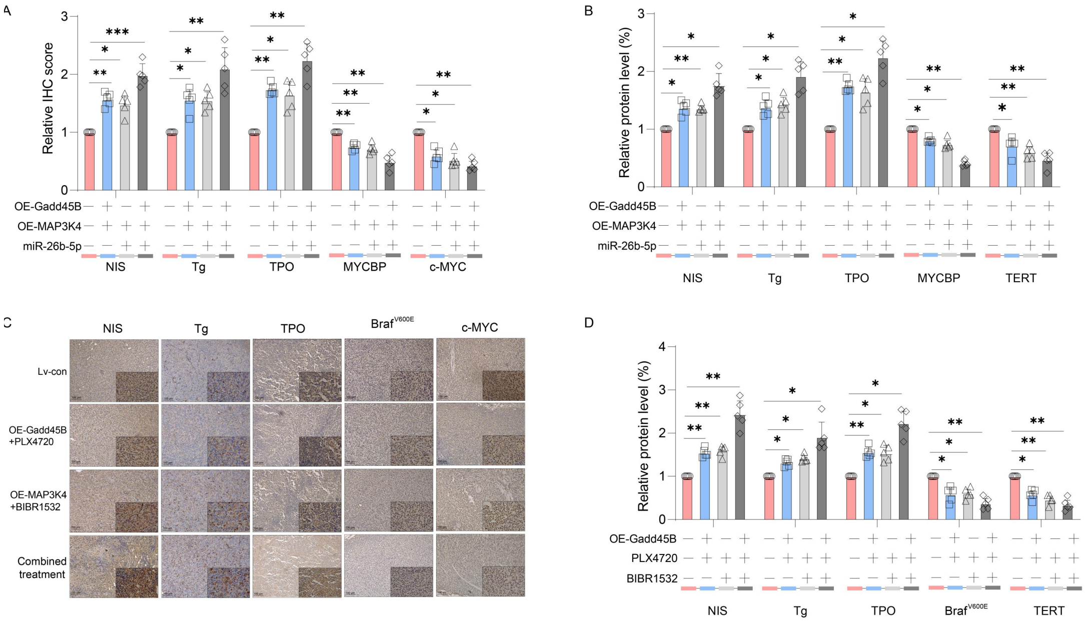

Figure S6.

(A) Quantitative analysis of IHC results from **Figure 6F**.

(B) Quantitative analysis of protein expression levels from **Figure 6G**.

(C) Representative IHC images depicting the expression of NIS, Tg, TPO, $BRAF^{V600E}$, and c-MYC in the indicated groups (scale bar: 100 μm).

(D) Quantitative analysis of protein expression levels from **Figure 6N**. Data are shown as mean ± SD. Statistical significance:ns: not significant, **p* < 0.05, ***p* < 0.01, ****p* < 0.001.

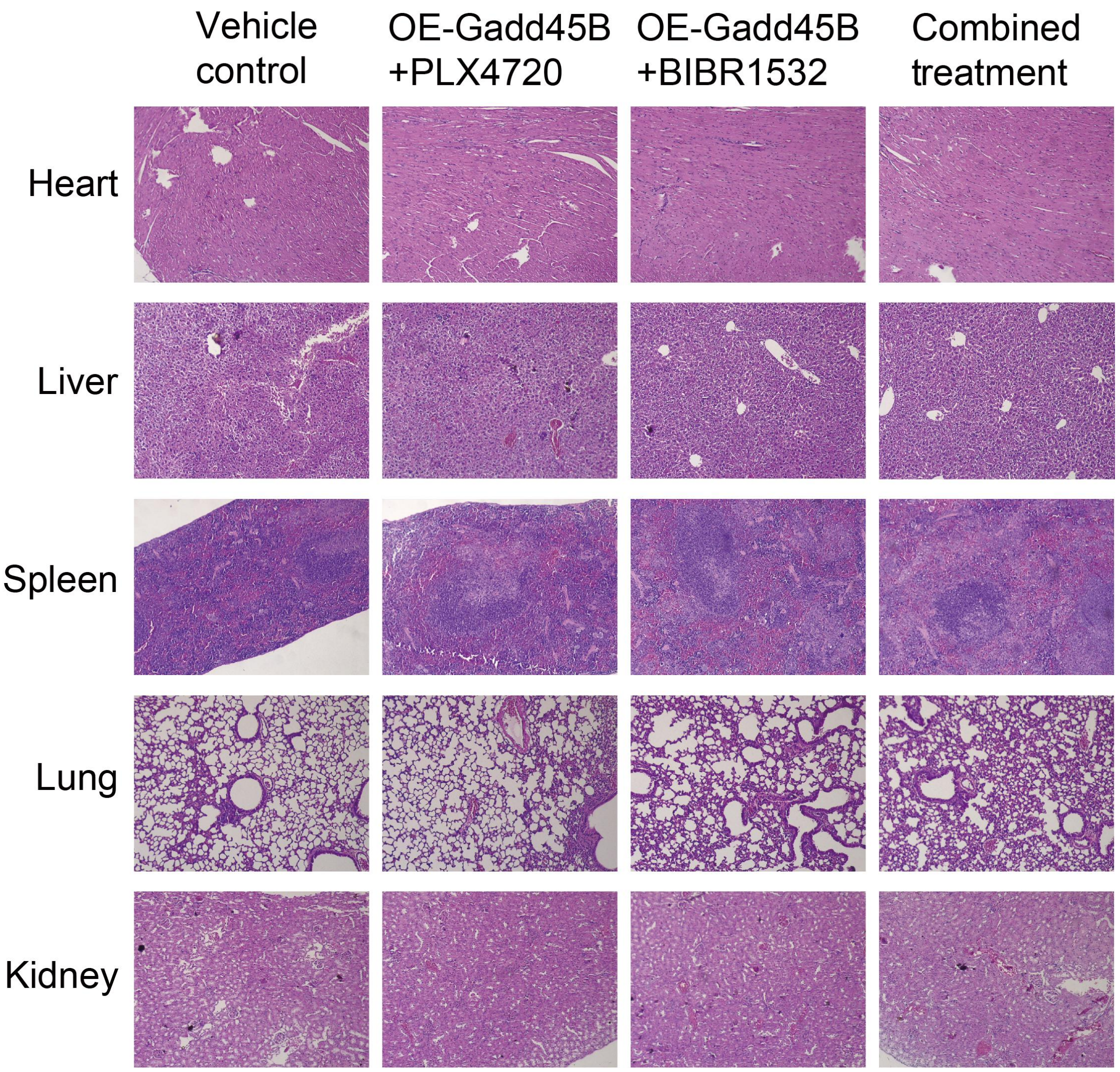

Figure S7

Representative H&E staining images of normal organs (heart, liver, kidney, spleen, lung) from mice in the indicated treatment groups (*n*=3; scale bar: 200 μm).

Table S1. Comparison of clinical characteristics and general information between RAIR-DTC and non-RAIR-DTC patients [*n* (%)]

Characteristics	Total（*n*=121）	non-RAIR-DTC（*n*=90）	RAIR-DTC（*n*=31）	χ^2	*p*
Gender				0.049	0.824
Female	80(66.12)	59(65.56)	21(67.74)		
Male	41(33.88)	31(34.44)	10(32.26)		
Age(years)				0.525	0.469
≤60	48(39.67)	34(37.78)	14(45.16)		
＞60	73(60.33)	56(62.22)	17(54.84)		
Diameter(cm)				0.376	0.539
≤1	72(59.5)	55(61.11)	17(54.84)		
＞1	49(40.5)	35(38.89)	14(45.16)		
Histological grade				26.749	<0.001
I/I-II/II	64(52.89)	60(66.67)	4(12.9)		
II-III/III	57(47.11)	30(33.33)	27(87.1)		
Tumor margin				45.157	<0.001
Sharply demarcated	70(57.85)	68(75.56)	2(6.45)		
Infiltrative growth	51(42.15)	22(24.44)	29(93.55)		
Perineural invasion				4.104	0.043
Absent	58(47.93)	48(53.33)	10(32.26)		
Present	63(52.07)	42(46.67)	21(67.74)		
T				0.155	0.694
Tis-T1	70(57.85)	53(58.89)	17(54.84)		
T2-T4	51(42.15)	37(41.11)	14(45.16)		
N				0.089	0.765
N0	48(39.67)	35(38.89)	13(41.94)		
N1, N2	73(60.33)	55(61.11)	18(58.06)		
M				2.602	0.107
M0	119(98.35)	90(100)	29(93.55)		
M1	2(1.65)	0(0)	2(6.45)		
Gadd45B				18.638	<0.001
L	40(33.06)	20(22.22)	20(64.52)		
H	81(66.94)	70(77.78)	11(35.48)		

Table S2. Univariate and multivariate analysis of factors associated with RAIR-DTC

Characteristics	*β*	*SE*	*p*	*OR*	95% *CI*
Gender (Male *vs*. Female)	-0.098	0.444	0.824	0.906	0.380-2.162
Age (>60 years *vs*. ≤60 years)	-0.305	0.421	0.469	0.737	0.323-1.684
Diameter (>1 cm *vs*. ≤1 cm)	0.258	0.421	0.540	1.294	0.567-2.952
Histological grade (II-III/III *vs*. I/I-II/II)	2.603	0.581	<0.001	13.500	4.327-42.119
Tumor margin (Infiltrative growth *vs*. Sharply demarcated)	3.803	0.771	<0.001	44.818	9.887-203.159
Perineural invasion (Present *vs*. Absent)	0.875	0.438	0.046	2.400	1.016-5.668
T (T2-T4 *vs*. Tis-T1)	0.165	0.420	0.694	1.180	0.518-2.685
N (N1, N2 *vs*. N0)	-0.127	0.423	0.765	0.881	0.384-2.020
M (M1 *vs*. M0)	15.060	747.965	0.984	>999.999	<0.001->999.999
Gadd45B expression (High *vs*. Low)	-1.851	0.453	<0.001	0.157	0.065-0.382

Logistic Regression Analysis

A one-way logistic regression analysis was conducted using the following independent variables: **Gender**, **Age**, **Diameter**, **Histological grade**, **Tumor margin**, **Perineural invasion**, **T (Tumor stage)**, **N (Nodal stage)**, **M (Metastatic stage)**, and Gadd45B expression levels.

Significant factors influencing RAIR occurrence ($p < 0.05$) included histological grade, tumor margin, perineural invasion, and Gadd45B expression.

These significant factors were included as independent variables in a stepwise multivariate logistic regression analysis.

Table S3. All antibodies used in this research.

Antibodies	Dilution	Source
Gadd45B	0.5 μg/mL	Invitrogen
MAP3K4	1:1000	Proteintech
MYCBP	1:1000	Proteintech
c-MYC	1:1500	Proteintech
MEK	1:5500	Proteintech
ERK	1:2500	Proteintech
p-MEK	1:1500	Proteintech
p-ERK	1:2500	Proteintech
TERT	1:1500	Proteintech
Sodium iodide symporter (NIS)	1:1000	Proteintech
Tg	1:1000	Proteintech
TPO	1:1500	Novus Biologicals
$BRAF^{V600E}$	1:1000	Invitrogen
SP1	1:2500	Proteintech
GAPDH	1:55000	Proteintech
HRP-conjugated Goat Anti-Rabbit IgG(H+L)	1:8000	Proteintech
HRP-conjugated Goat Anti-Mouse IgG(H+L)	1:8000	Proteintech
